# Efficacy and safety of telitacicept as an add-on therapy for relapsing lupus nephritis: a retrospective cohort study

**DOI:** 10.3389/fimmu.2026.1826829

**Published:** 2026-05-22

**Authors:** Cui Wang, Xue Bai, Jiawen Li, Jie Zhao, Yarui Zhang, Xuehong Lu, Qiaoyan Guo

**Affiliations:** Department of Nephrology and Rheumatology, The Second Hospital of Jilin University, Changchun, Jilin, China

**Keywords:** efficacy, lupus nephritis, relapse, safety, telitacicept

## Abstract

**Aim:**

This study aims to evaluate the efficacy and safety of telitacicept addition to standard therapy in adults with relapsing lupus nephritis (LN).

**Methods:**

From 2021 to 2024, patients with relapsing LN were identified and divided into two groups based on telitacicept administration. Laboratory indicators, renal remission status, Systemic Lupus Erythematosus Disease Activity Index 2000 (SLEDAI-2K) scores, glucocorticoid dosages, and the incidence of renal flares were evaluated. Multivariate regression was used to assess the baseline predictors of complete renal remission (CRR) and adverse events (AEs) were recorded.

**Results:**

Compared to the standard therapy group (n=20), the proportion of patients who achieved a CRR and the primary efficacy renal response were significantly increased in the telitacicept group (n=20) at 6, 9, and 12 months, while the reduction rates of 24-hour urinary protein from baseline at 1, 3, and 9 months were significantly higher. The telitacicept group showed notable improvement in treatment responses in the median SLEDAI-2K score and glucocorticoid dose. Multivariate Cox regression analysis revealed that add-on telitacicept was associated with achieving early CRR. At the end of the follow-up period, the cumulative relapse rate in the telitacicept group was significantly lower than that in the standard treatment group, with no increase in the incidence of AEs.

**Conclusions:**

As an add-on therapy, telitacicept is associated with early disease remission in patients with relapsing LN with reduced disease activity, lower glucocorticoid dosage, and fewer relapses. It also showed a favorable safety profile.

## Graphical Abstract

**Figure:**
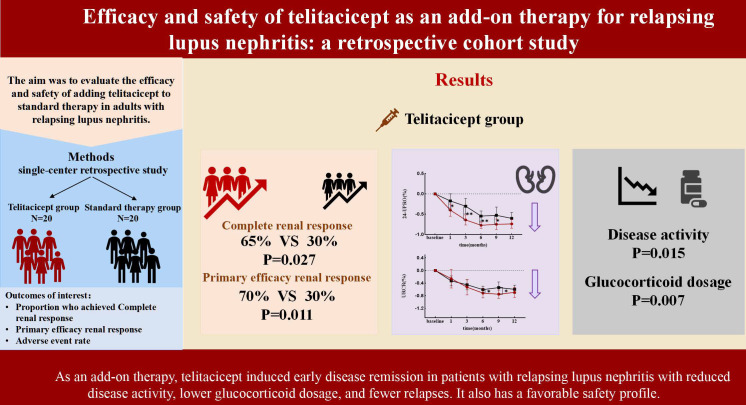

## 1 Introduction

Lupus nephritis (LN) is the most common complication of systemic lupus erythematosus (SLE); approximately 30%–60% of adult patients and up to 70% of pediatric patients with SLE develop LN (1), and approximately 10% of patients with LN may progress to end-stage kidney disease (ESKD) within 10 years (2). Because each episode or relapse of LN contributes to chronic kidney disease (CKD) progression, prevention of flares is crucial (3). Although considerable advances have been made in LN treatment to date, glucocorticoids and immunosuppressants carry severe short- and long-term toxicities. Moreover, a subset of patients shows no response to standard regimens or frequent disease flares (4). Notably, for relapsing LN, the combined use of novel biological agents reduces drug toxicity, lowers relapse risk, slows renal progression, and improves long-term patient and renal survival (5).

B cells play a crucial role in the pathogenesis of LN, with the loss of central and peripheral immune tolerance leading to the proliferation of autoreactive B cells (6). B-lymphocyte stimulator (BLyS) and A proliferation-inducing ligand (APRIL) regulate B cell activation, differentiation, and maturation and promote the production of autoantibodies. Patients with SLE and LN exhibit a notable increase in BLyS and APRIL levels, inhibition of which helps to alleviate the clinical symptoms of LN (7–9). Telitacicept is a novel monoclonal antibody formed by fusing the gene fragment of the TACI protein (a common ligand for BLyS and APRIL) with the crystallizable fragment (Fc) of the human IgG1 protein. It prevents the interaction between BLyS/APRIL and its receptors by recognizing BLyS and APRIL, thereby inhibiting the activation of B and plasma cells (10).

A phase IIb clinical trial of telitacicept for the treatment of SLE demonstrated that the efficacy achieved by the telitacicept group at week 48 was significantly superior to that of the placebo group (71.0%–75.8% vs. 33.9%, both p < 0.001). Additionally, the time to the first severe flare in the telitacicept group was significantly longer than that in the placebo group (148–227 days vs. 113 days, both p < 0.05), with comparable safety profiles (11). The latest phase III clinical trial revealed that the SLE responder index (SRI-4) remission rate at week 52 in the telitacicept-treated SLE group was 67.1% compared with 32.7% in the standard treatment group (p < 0.001) (12). In terms of LN treatment, studies have found that telitacicept can significantly improve disease activity and proteinuria levels in patients with nephrotic syndrome-associated LN (13). A real-world retrospective cohort study demonstrated that adding telitacicept to the standard treatment significantly enhanced clinical remission rates and improved the prognosis of patients with class III–V LN (14). Furthermore, in patients with LN, telitacicept can reduce the disease severity and daily glucocorticoid intake, while maintaining safety and improving hypocomplementemia (15, 16). However, it remains unclear whether telitacicept offers greater advantages in patients with relapsed LN.

Therefore, we conducted a single-center retrospective observational study to extend previous research by evaluating the renal protective effects of telitacicept in relapsed LN, with the aim of assessing the efficacy and safety of add-on telitacicept therapy for the treatment of recurrent LN.

## 2 Methods

### 2.1 Study design and patients

**Figure 1:**
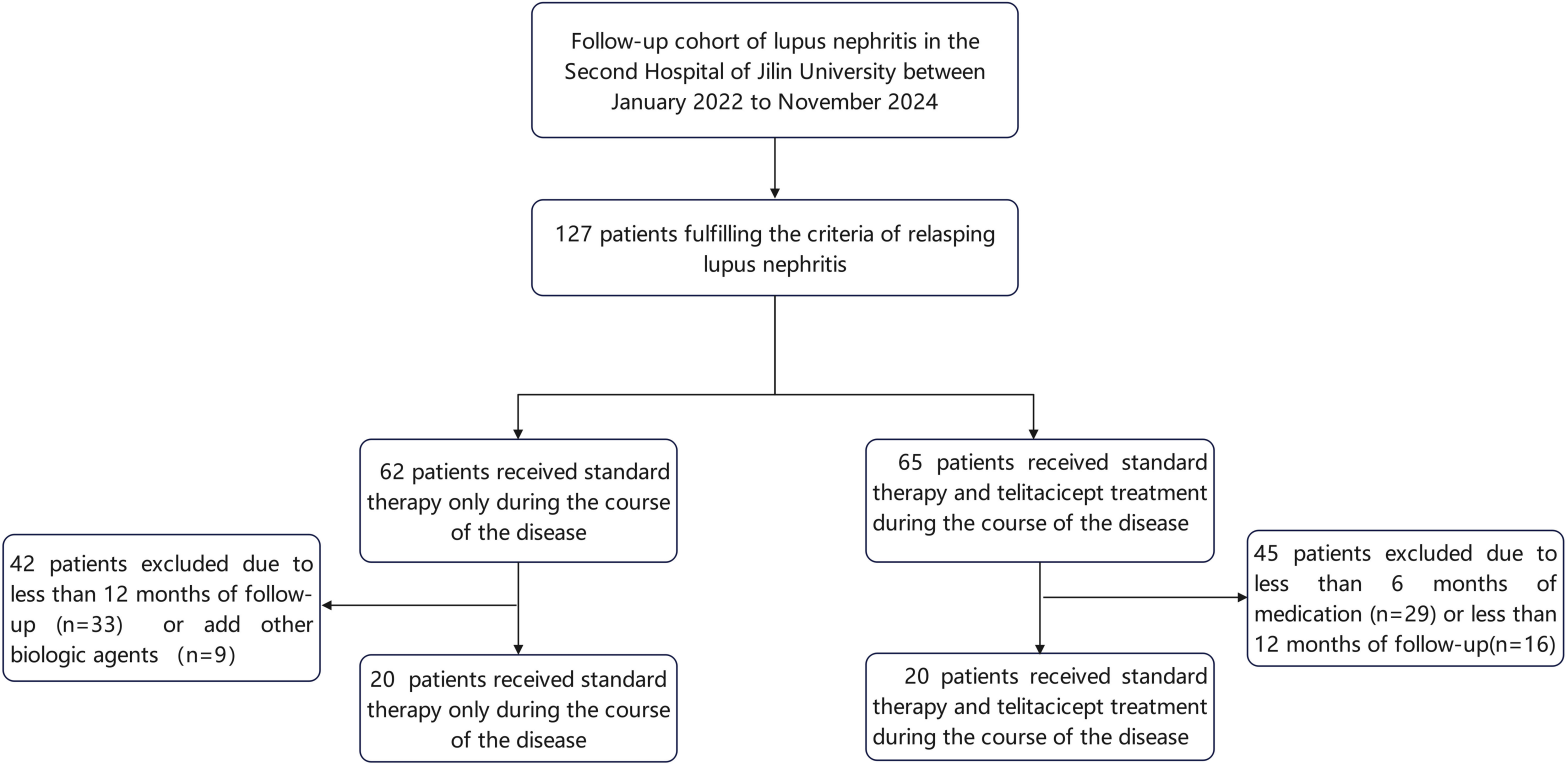
Research flowchart of the study.

This was a single-center retrospective study. Patients from the Nephrology Departments of the Second Hospital of Jilin University between January 2021 and October 2024 were enrolled in the study (**Figure 1**) and divided into the telitacicept treatment group (telitacicept plus standard therapy) and the standard therapy group. The inclusion criteria were as follows: (1) Aged 18 to 70 years (inclusive); (2) fulfilling the 1997 American College of Rheumatology (ACR) classification criteria for SLE (17); (3) confirmed diagnosis of LN via renal biopsy performed in accordance with the recommendations of the 2024 Kidney Disease: Improving Global Outcomes (KDIGO) Clinical Practice Guidelines, or persistent abnormal urinary test results supporting a diagnosis of LN (18); (4) a follow-up duration of more than 12 months with complete and intact case data; (5) receiving a stable standard treatment regimen for LN (patients in the telitacicept group received regular telitacicept administration at a dose of 160 mg once weekly for a minimum of 6 months); and (6) meeting the diagnostic criteria for relapsing LN. LN relapse was defined as follows: Recurrence of proteinuria, where patients in complete remission developed a 24-h urinary protein quantification ≥ 1.0 g; patients in partial remission had an increase in 24-h urinary protein excretion of more than 2.0 g, with or without exacerbated hematuria (doubled red blood cell count in urinary sediment, red blood cell count elevated from < 5/high-power field [HPF] to > 10/HPF, or the emergence of red blood cell casts and leukocyte casts); or patients without remission had a twofold increase in urinary protein compared with baseline levels. An elevated serum creatinine (Scr) level was defined as a 50% increase in Scr from the prior normal baseline, or an increase of ≥ 30% in Scr from the prior abnormal baseline (19).

The exclusion criteria were as follows: (1) Patients with severe renal impairment (estimated glomerular filtration rate [eGFR] ≤ 15 mL/min/1.73 m²) or those requiring long-term renal replacement therapy at baseline; (2) receipt of other B-cell targeted therapies (e.g., rituximab, belimumab) within 1 month prior to the start of observation/treatment or during the observation period; (3) comorbidity with other primary or secondary glomerular diseases (e.g., primary immunoglobulin A (IgA) nephropathy, diabetic nephropathy); (4) comorbidity with severe organic diseases involving other organs (e.g., the heart, liver, or nervous system); (5) comorbidity with active infection or malignant tumors; and (6) pregnant women, women planning pregnancy in the near term, and lactating women.

Telitacicept was administered subcutaneously at doses of 160mg, with continuous treatment for at least 6 months. The telitacicept group receives telitacicept in combination with standard therapy. Standard therapy consisted of glucocorticoids combined with immunosuppressants. All patients are treated with glucocorticoids (0.5 - 1.0 mg/kg/day, with slow tapering over 4–6 weeks and long-term maintenance at less than 5.0 mg/day, with the possibility of discontinuation if conditions permit) and hydroxychloroquine (<5 mg/kg/day) as the foundational treatment drugs. When necessary, immunosuppressants should be used in combination, such as cyclophosphamide (CTX,500 mg intravenously every two weeks for a total of six doses, or 0.5 to 1 g/m² intravenously once a month for 6 months), mycophenolatemofetil (MMF,1.0 - 2.0 g/day, with a maintenance dose of 0.5–1 g/day), tacrolimus(TAC, 0.05-0.1 mg/kg/day, targeting trough levels of 4–7 ng/mL),cyclosporine A (CsA, 3–5 mg/kg/day, targeting trough levels of 100–150 ng/mL), or combinations thereof. Clinical doctors make individualized selections for patients without any artificial intervention from researchers. This study was conducted in accordance with the Declaration of Helsinki and approved by the Scientific Research Ethics Committee of the Second Hospital of Jilin University (Approval No.2024-381).

### 2.2 Data collection

Data from the first administration of telitacicept or standard treatment following the confirmation of LN relapse were defined as baseline. Clinical data were collected at baseline, 1, 3, 6, 9, and 12 months, including sex, age, hemoglobin (Hb), serum albumin (Alb), Scr, eGFR, complement C3, complement C4, 24-h urinary total protein (24h-UTP), urinary protein-to-creatinine ratio (UPCR), positive rate of antinuclear antibody (ANA) (ANA titer ≥ 1:80), positive rate of anti-double-stranded DNA (anti-ds-DNA) antibodies, SLE Disease Activity Index 2000 (SLEDAI-2K) activity score (20), and daily glucocorticoid dosage. In addition, the number of LN flares at baseline was documented.

### 2.3 Endpoints

The primary endpoint was the renal remission rate during follow-up, including complete renal response (CRR) and primary efficacy renal response (PERR). CRR was defined as 24h-UTP< 0.5 g or UPCR < 500 mg/g (50 mg/mmol), serum Alb ≥ 35 g/L, and an eGFR that was no less than 10% below the pre-flare value or ≥ 90 ml/min*1.73 m^2^, and no rescue therapy. PERR was defined as UPCR < 0.7 g/g(70 mg/mmol), an eGFR that was no worse than 20% below the pre-flare value or ≥ 60 mL per min per 1.73 m^2^, and no rescue therapy (18). Secondary efficacy evaluation included 24h-UTP, UPCR, Alb, SCr, eGFR, complement C3, complement C4, anti-ds-DNA levels, IgG, IgA, IgM, SLEDAI-2K score, and daily glucocorticoid dosage.

Adverse events (AEs) at each follow-up visit were documented and included leukocytopenia, respiratory tract infections, injection site rashes, urinary tract infections, and fever.

### 2.4 Statistical analysis

Statistical analyses were performed using IBM SPSS Statistics for Windows, version 26.0, R (version 4.4.3; IBM, Armonk, NY, USA), and GraphPad Prism 9 (GraphPad, Boston, MA, USA).Continuous variables are expressed as the mean ± standard deviation or median (25%–75% interquartile range), and comparisons between groups were performed using t-test or Mann–Whitney U test according to whether the data distribution was normal or not. The categorical data were summarized as frequencies (n) with percentages (%), and the χ2 test was used for comparisons between groups. Survival analysis was performed to evaluate the time to Cumulative renal remission (CRR) or LN relapse. Survival time was calculated from the date of treatment initiation to the date of CRR or LN relapse, with censoring as the end of follow-up without CRR or LN relapse. The Kaplan-Meier method was used to estimate survival probabilities, and differences were compared by the Log-rank test. Results are expressed as hazard ratios with 95% confidence intervals. Patients who did not achieve CRR by the end of follow-up were considered censored. We define censoring as the end of follow-up without CRR. To assess the potential for informative censoring, we compared baseline characteristics between patients who achieved CRR (non-censored) and those who were censored, using t-test (or Mann-Whitney U test) for continuous variables and chi-square (or Fisher’s exact) test for categorical variables (**Supplementary Table 1**). P-values < 0.05 were considered statistically significant.

## 3 Results

### 3.1 Baseline characteristics

Forty patients were enrolled in the study (**Figure 1**). There were no significant differences in baseline clinical characteristics, laboratory indicators, SLEDAI-2K, daily glucocorticoid dosage, or therapy regimen between the two groups (p > 0.05) (**Table 1**). A total of 32 patients underwent renal biopsy. In the telitacicept group (n = 13), two patients had class III/IV LN, eight had class III/IV + V LN, and three had class V LN. In the standard therapy group (n = 19), there were two cases of class II LN, five of class III/IV LN, ten of class III/IV + V LN, and two of class V LN. The pathological types were comparable between the two groups.

**Table 1:** Comparison of clinical characteristics and medications in patients treated with or without telitacicept at baseline.

Characteristics	Telitacicept (n=20)	Standard therapy (n=20)	P
Female, n (%)	20 (100%)	20 (100%)	–
age (y)	34.40 ± 14.96	31.15 ± 10.81	0.436
LN course (y)	3.00 (1.00, 7.00)	6.00 (4.00, 8.00)	0.064
relapse frequency			0.723
<2	14 (70%)	15 (75%)	
≥2	6 (30%)	5 (25%)	
Hypertension (n%)	8 (40.0%)	9 (45.0%)	0.749
Hb (g/L)	115.45 ± 25.91	111.05 ± 23.70	0.578
PLT (10^9/L)	204.00 ± 87.15	209.00 ± 87.47	0.857
WBC (10^9/L)	7.30 (5.73,8.38)	5.80 (4.65,7.90)	0.133
Alb (g/L)	32.67 ± 7.73	28.08 ± 7.81	0.069
Scr (umol/L)	63.50 (53.25,78.50)	83.50 (52.50,191.00)	0.172
BUN (mmol/L)	6.78 (4.83,8.80)	7.38 (6.22,13.70)	0.194
eGFR (ml/min/1.73m^2)	103.50 (85.10,123.50)	81.25 (29.30,117.15)	0.130
Urine RBC (/HP)	7.45 (2.83, 21.78)	8.50 (2.50, 31.6)	0.607
24h-UTP (g/24h)	2.30 (1.73, 3.30)	3.47 (1.60, 7.58)	0.344
UPCR (mg/mmol)	294.50 (169.14, 395.42)	394.64 (225.83, 924.76)	0.076
Positive-Anti-Sm (n, %)	6 (30.0%)	11 (55.0%)	0.110
Positive-ANA≧1:320[n (%)]	19 (95.0%)	20 (100%)	–
Positive-anti ds-DNA[n (%)]	12 (60%)	12 (60%)	–
C3 (mg/dL)	54.42 ± 20.95	43.30 (31.48, 65.50)	0.387
C4 (mg/dL)	10.79 ± 4.82	7.00 (5.35, 13.63)	0.265
IgG (g/L)	12.96 ± 5.02	11.35 (7.18, 14.00)	0.372
IgA (g/L)	2.88 (1.87, 3.80)	2.15 (1.56, 3.52)	0.250
IgM (g/L)	0.92 (0.63, 1.20)	0.80 (0.45, 1.15)	0.358
SLEDAI-2K	13.80 ± 2.86	14.75 ± 3.73	0.371
Disease activity			0.496
Mild	0 (0%)	0 (0%)	–
Moderately	7 (35%)	5 (25%)	–
Severe	13 (65%)	15 (75%)	–
glucocorticoid dosage (mg)/day	30.00 (30.00, 42.50)	40.00 (30.00, 50.00)	0.076
Pathology ISN/RPS class (%)	n=13	n=19	0.076
II	0 (0%)	2 (10.0%)	
III/IV	2 (10.0%)	5 (25.0%)	
III/IV+V	8 (40.0%)	10 (50.0%)	
V	3 (15.0%)	2 (10.0%)	
no biopsy	7 (35.0%)	1 (5.0%)	
Therapy			0.612
MMF	10 (50.0%)	9 (45.0%)	–
CTX	2 (10.0%)	5 (25.0%)	–
TAC	4 (20.0%)	2 (10.0%)	–
MMF+TAC	4 (20.0%)	3 (15.0%)	–
No	0	1 (5.0%)	–

LN, lupus nephritis; Hb, hemoglobin; PLT, platelet; WBC, white blood cell; Alb, albumin; UA, uric acid; BUN, blood urea nitrogen; Scr, serum creatinine; eGFR, estimated glomerular filtration rate; 24h-UTP, 24-h urinary total protein; UPCR, Urine protein creatinine rate; IgA, immunoglobulin A; IgG, immunoglobulin G; IgM, immunoglobulin M; SLEDAI-2K, The Systemic Lupus Erythematosus Disease Activity Index 2000;GC, glucocorticoids; MMF, mycophenolate mofetil; CTX, cyclophosphamide; TAC, Tacrolimus.

### 3.2 Efficacy outcomes

#### 3.2.1 Renal outcome

**Figure 2:**
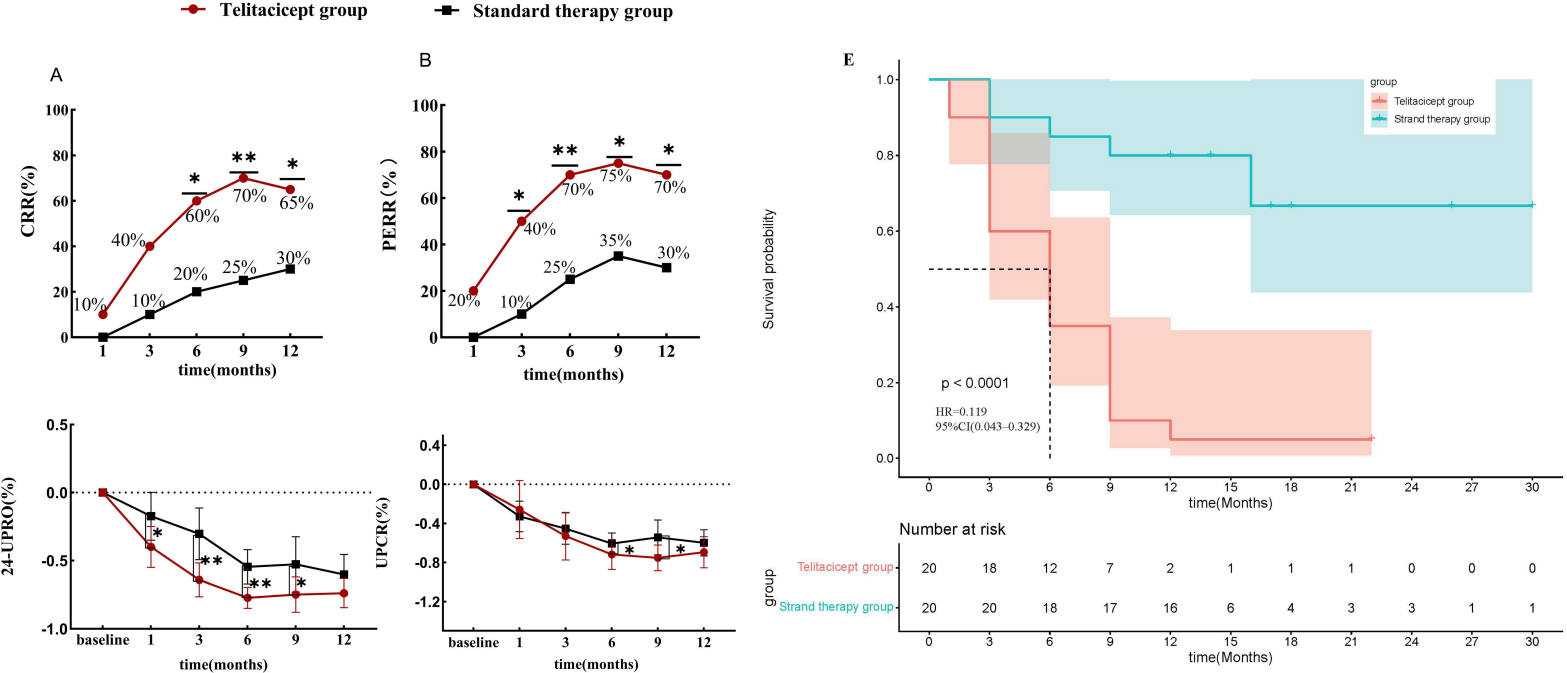
Efficacy of telitacicept on renal outcome. Comparison of the proportions of patients with relapse LN achieving CRR **(A)** and PERR **(B)**, comparison of the percentage reduction from baseline in 24h-UTP **(C)** and UPCR **(D)** between the two groups during follow-up, and cumulative CRR rate during follow-up between the two groups (Kaplan–Meier survival curve) **(E)**. *p < 0.05, **p < 0.01. LN: lupus nephritis; CRR: complete renal remission; PERR: primary efficacy renal response; 24h-UTP: 24-h urinary total protein; UPCR: Urine protein creatinine rate.

In terms of renal remission, the CRR rates in the telitacicept group at 1, 3, 6, 9, and 12 months were 10.0% (2/20), 40.0% (8/20), 60.0% (12/20), 70.0% (14/20), and 65.0% (13/20), respectively, while those in the standard treatment group at the corresponding time points were 0.0% (0/20), 10.0%(2/20), 20.0%(4/20), 25.0%(5/20), and 30.0%(6/20), respectively. The CRR rates of the telitacicept group were significantly higher than those of the standard treatment group at 6 (p = 0.024), 9 (p = 0.004), and 12 months (p = 0.027) (**Figure 2A**).

The PERR rates in the telitacicept group at 1, 3, 6, 9, and 12 months were 20.0% (4/20), 40.0% (8/20), 70.0% (14/20), 75.0% (15/20), and 70.0% (14/20), respectively, while those in the standard treatment group at the corresponding time points were 0.0% (0/20), 10.0% (2/20), 25.0% (5/20), 35.0% (7/20), and 30.0% (6/20), respectively. The PERR rates in the telitacicept group were significantly higher than those in the standard treatment group at 3 (p = 0.016), 6 (p = 0.004), 9 (p = 0.011), and 12 months (p = 0.011) (**Figure 2B**).

By the end of the follow-up, using CRR as the endpoint, the time to first CRR attainment during the follow-up was collected. A total of 24 patients (62.5%) achieved the first complete renal remission (CRR). In the telitacicept group, 19 out of 20 patients (95.0%) reached CRR, compared with 5 out of 20 patients (25.0%) in the control group. Kaplan–Meier survival analysis revealed that the cumulative complete remission rate of the telitacicept group was significantly higher than that of the standard treatment control group (log-rank p < 0.0001,hazard ratio [HR]:0.1196.120, 95% confidence interval [CI]: 0.043,0.329 (**Figure 2E**).

In the telitacicept group, the median 24h-UTP significantly decreased from 2.30 g at baseline to 0.68 g at 3 months, 0.43 g at 6 months, and 0.56 g at 12 months. In contrast, in the standard treatment group, the median 24h-UTP significantly decreased from 3.47 g at baseline to 1.78 g at 3 months, 1.07 g at 6 months, and 0.79 g at 12 months. The 24h-UTP levels in the telitacicept group were significantly lower than those in the standard treatment group at 1 (p = 0.023), 3 (p = 0.002), 6 (p = 0.004), 9 (p = 0.006), and 12 months (p = 0.038) (**Supplementary Table 2**). The percentage reduction in 24h-UTP from baseline in the telitacicept group was significantly higher than that in the standard treatment group at 1 (p = 0.021), 3 (p = 0.004), 6 (p = 0.004), and 9 months (p = 0.048) (**Figure 2C**). Similarly, the percentage reduction in UPCR from baseline was significantly higher in the telitacicept group than in the standard treatment group at 6 months (p = 0.029) and 9 months (p = 0.045) (**Figure 2D**). No significant differences in urinary red blood cell counts and eGFR were observed between the two groups (**Supplementary Table 2**).

### 3.3 Serologic features and activity assessment

**Figure 3:**
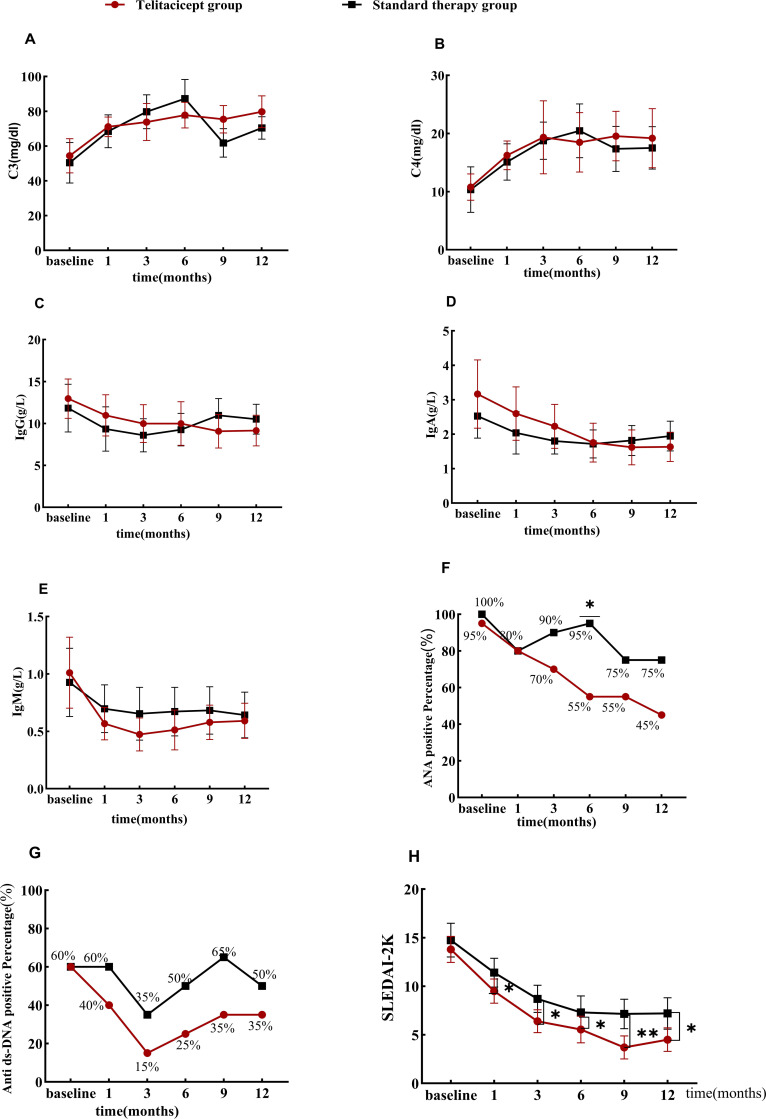
Efficacy of telitacicept on serologic features and activity assessment. Comparisons of the levels of C3 **(A)**, C4 **(B)**, IgG **(C)**, IgA **(D)**, IgM **(E)**, ANA positive **(F)**, anti-ds-DNA antibody positive **(G)**, SLEDAI-2K score **(H)** at baseline and months 1, 3, 6, 9, and 12 between two groups. *p < 0.05, **p < 0.01. IgA, immunoglobulin A; IgG, immunoglobulin G; IgM, immunoglobulin M.

At 6 months, the telitacicept group showed a significantly greater reduction in ANA levels compared with the standard therapy group (55% vs. 95%, p = 0.011) (**Figure 3F**). There was no significant difference in anti-dsDNA antibody levels between the two groups (**Figure 3G**). The complement levels increased in both groups, but the difference was not statistically significant (**Figures 3A, B**). The IgG, IgA, and IgM levels decreased in both groups, but without statistical differences (**Figures 3C–E**). Disease activity (SLEDAI-2K scores) decreased significantly in both groups, with the telitacicept group showing a more substantial reduction at 1 month (p = 0.046), 3 months (p = 0.012), 6 months (p = 0.048), 9 months (p = 0.003), and 12 months (p = 0.015) (**Figure 3H**).

### 3.4 Factors associated with achieving a CRR

Univariate Cox regression analysis revealed that the add-on telitacicept therapy [HR: 6.897, 95% CI (2.493, 19.078), p < 0.001], LN course [HR: 0.845, 95% CI (0.734, 0.973), p = 0.020], Scr [HR: 0.990, 95% CI (0.980, 1.000), p = 0.042], eGFR [HR: 1.018, 95% CI (1.005, 1.031), p = 0.006], UA [HR: 0.995, 95% CI (0.991, 0.999), p = 0.014], BUN [HR: 0.885, 95% CI (0.792, 0.989), p = 0.031], Alb [HR: 1.077, 95% CI (1.018, 1.138), p = 0.009], 24h-UTP [HR: 0.775, 95% CI (0.625, 0.960), p = 0.020], and UPCR [HR: 0.998, 95% CI (0.996, 1.000), p = 0.023] influenced CRR. Variables with a P-value < 0.05 in univariate Cox regression were incorporated into multivariate regression, with grouping serving as the categorical covariate. Ultimately, the Telitacicept group [HR: 4.949, 95% CI (1.574, 15.564), p = 0.006] was associated with achieving an early CRR (**Table 2**).

**Table 2:** Baseline predictors of CRR by univariate and multivariate Cox analysis.

Variable	Univariate		Multivariate	
	HR (95%CI)	P	HR (95%CI)	P
Group				
standard therapy	1.000 (reference)		1.000 (reference)	
** telitacicept**	6.897 (2.493,19.078)	<0.001*	4.949 (1.574, 15.564)	0.006*
LN course	0.845 (0.734,0.973)	0.020*		
Scr	0.990 (0.980,1.000)	0.042*		
eGFR	1.018 (1.005,1.031)	0.006*		
BUN	0.885 (0.792, 0.989)	0.031*		
Alb	1.077 (1.018, 1.138)	0.009*		
24h-UTP	0.775 (0.625, 0.960)	0.020*		
UPCR	0.998 (0.996, 1.000)	0.023*		
UA	0.995 (0.991,0.999)	0.014*		

HR, hazard ratios; CI, confidence interval; Alb, albumin; UA, uric acid; BUN, blood urea nitrogen; SCr, serum creatinine; 24h-UTP, 24-h urinary total protein; UPCR, Urine protein creatinine rate; *P<0.05. Bold values indicate that are statistically significant (P < 0.05) in the multivariate Cox regression model.

### 3.5 Subgroup analysis of CRR

**Figure 4:**
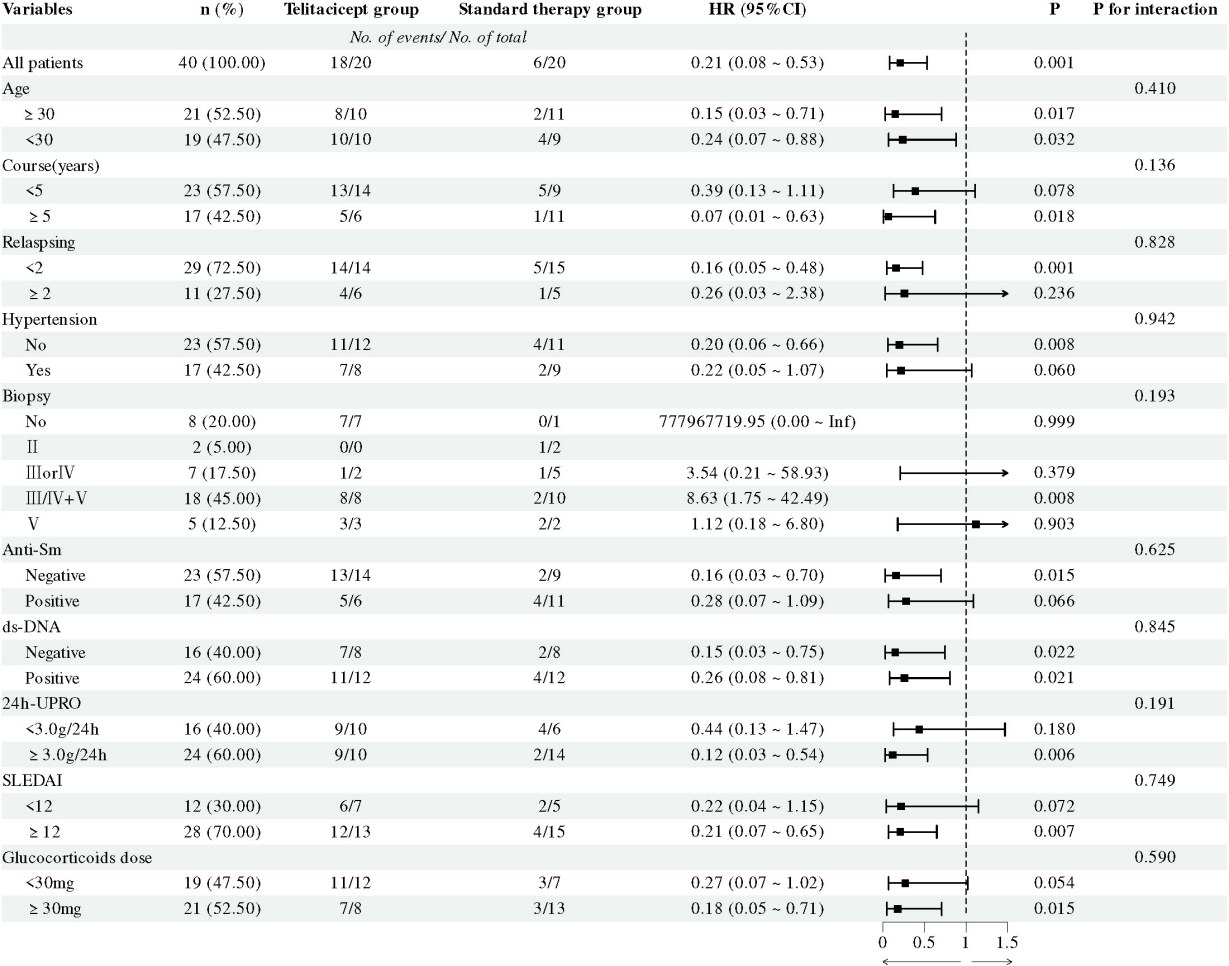
Subgroup analysis for achieving CRR between the two groups. CRR: complete renal remission.

The patients were classified based on their baseline clinical characteristics and laboratory indicators. We examined the interaction between baseline subgroup factors and treatment regimens, and the results showed no significant interactions among the subgroups (interaction p > 0.05). This indicates that the efficacy of telitacicept was consistent regardless of age, disease duration, number of relapses, pathological morphology, and other factors (**Figure 4**).

### 3.6 Glucocorticoid dosage and changes in immunosuppressive regimens

**Figure 5:**
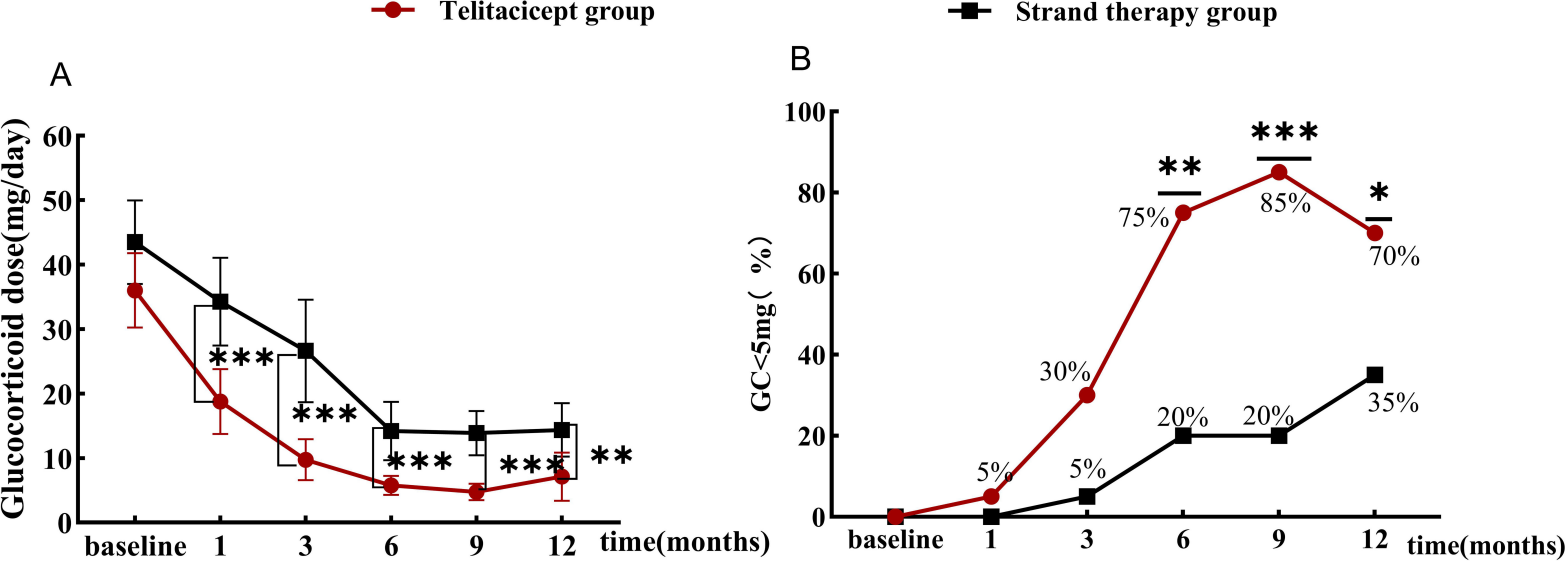
Efficacy of telitacicept for reducing the glucocorticoid dosage **(A)**. Comparison of the daily glucocorticoid dosage between the two groups **(B)**. Percentage of patients with ≤ 5 mg/day glucocorticoid between the two groups. *p < 0.05, **p < 0.01, ***p < 0.001.

In the telitacicept group, median glucocorticoid dose decreased from 30 mg at baseline to 7.5 mg at 3 months, 5.0 mg at 6 months, and 5.0 mg at 12 months. In contrast, in the standard treatment group, median glucocorticoid dose decreased from 40 mg at baseline to 25 mg at 3 months, 10 mg at 6 months, and 15 mg at 12 months. The glucocorticoid dose in the telitacicept group was significantly lower than in the standard treatment group at 1 (p < 0.001), 3 (p < 0.001), 6 (p < 0.001), 9 (p < 0.001), and 12 months (p = 0.007) (**Figure 5A**). Compared with the control group, the telitacicept group had more patients with ≤ 5 mg/day glucocorticoid at 6 months (75% vs. 20%, p = 0.002), 9 months (85% vs. 20%, p < 0.001), and 12 months (70% vs. 35%, p = 0.027) (**Figure 5B**).

Changes in immunosuppressive regimens were minimal. By month 6, after reaching the full dose, CTX was switched to other immunosuppressants. There were no statistically significant differences in immunosuppressants between baseline and follow-up periods (P > 0.05) (**Supplementary Table 2**).

### 3.7 Prognosis

**Figure 6:**
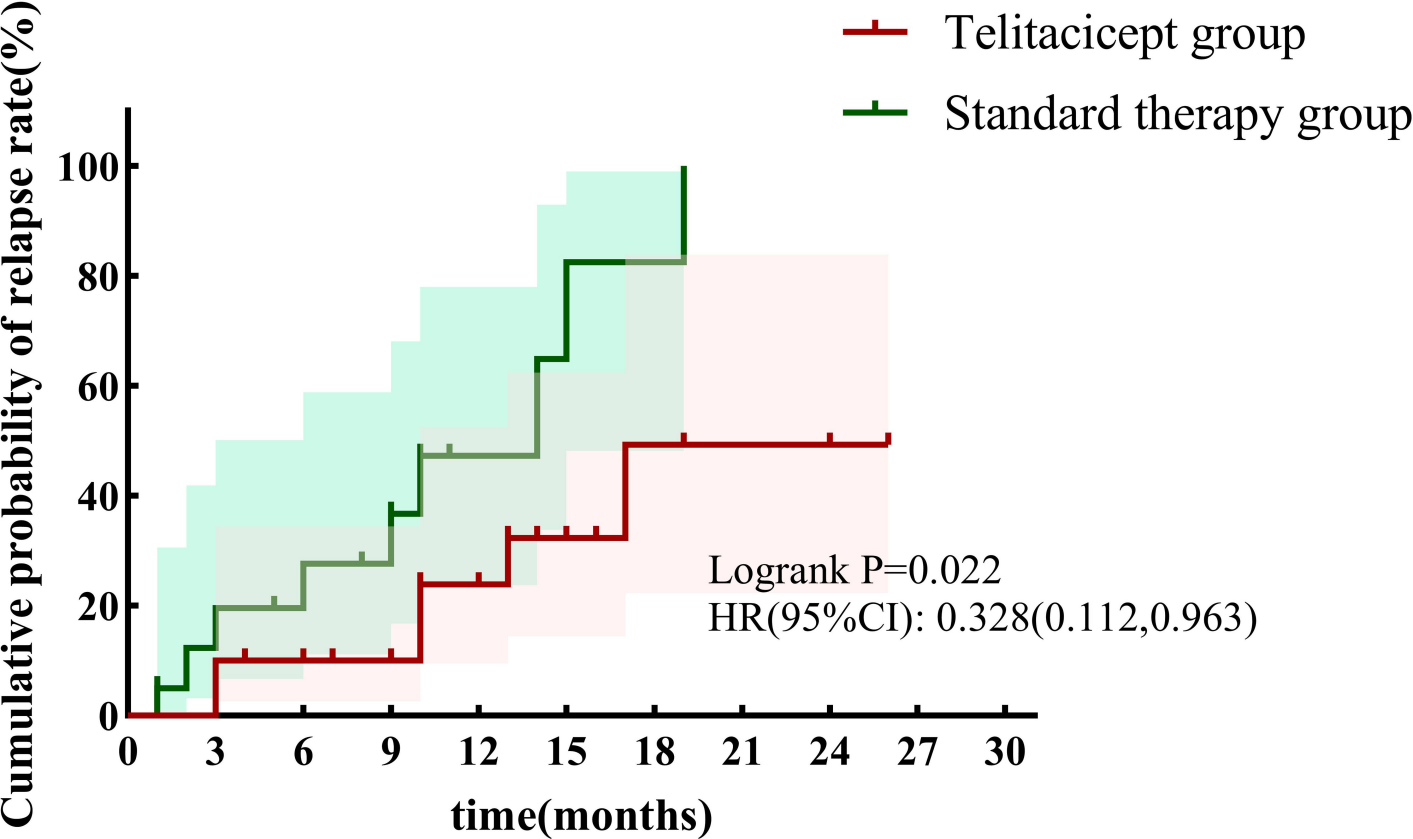
Cumulative relapse rate during follow-up between the two groups (Kaplan–Meier survival curve).

In patients with relapsing LN, 30.0% of those in the telitacicept group experienced relapse compared with 45% in the control group (p = 0.327). By the end of follow-up, using relapse as the endpoint, Kaplan–Meier survival analysis revealed that the cumulative relapse rate in the telitacicept group was significantly lower than in the standard treatment group [p = 0.022, HR: 0.328, 95% CI (0.112, 0.963)] (**Figure 6**).

### 3.8 Safety outcomes

During the 12-month follow-up, four patients (20.0%) in the telitacicept group experienced adverse reactions, including three cases (15.0%) of urinary tract infections and one case (5.0%) of rash. In the standard treatment group, seven patients (35.0%) reported adverse reactions, comprising four cases (20.0%) of pulmonary infections and three cases (15.0%) of urinary tract infections. There was no significant difference in the incidence of adverse reactions between the two groups (p = 0.479), and no severe adverse reactions were observed.

## 4 Discussion

The disease course of LN is characterized by flares of activity alternating with periods of quiescence; renal flares and LN relapse often portend poor renal survival, and the prevention of disease flares remains challenging. Previous studies have suggested that telitacicept, a fusion protein that simultaneously inhibiting Blys and APRIL to regulate abnormal B cells, can reduce serological relapse of LN; however, its impact on renal relapse remains unclear (15). Previously, there was a lack of research on telitacicept in specific patient populations with LN relapse. In this retrospective cohort study, we found that 6 months of add-on therapy with telitacicept could enhance renal remission, reduce disease activity, decrease glucocorticoid dosage, lower disease relapse rates, and do not increase the incidence of AEs in patients with relapse of LN, which may suggest its efficacy and safety.

In the current study, we found that compared with the standard treatment, add-on therapy with telitacicept reduced the levels of 24h-UTP and UPCR in patients with relapsed LN. The 24h-UTP decreased to below 0.5 g at 6 months. It demonstrated superior efficacy in achieving renal remission, with significant differences in PERR rates between the two groups observed at 3 months. At 6 months, the CCR rates and PERR rates were 70% and 75%, respectively, while at 12 months, they were 65% and 70%, respectively. Multivariate analysis also confirmed that telitacicept was a favorable factor for the attainment of CRR. Similarly, in a self-controlled before-after study, Jin et al. found that renal function improved in 34 patients with LN after treatment with telitacicept, with significant reductions in 24h-UTP and UPCR at 24 weeks, both the CRR and PERR exceeded 70% (21). Huang et al. reported that the CRR rates reached 73.3% in 30 patients treated with telitacicept (16). In a study targeting patients with class III–V LN, telitacicept demonstrated remarkable efficacy in improving urinary protein levels, with CRR rates of 64.4% and 80.8% at 6 and 12 months, respectively, in the telitacicept group (14). Interestingly, a real-world study by Liu et al. reported that in 67 patients with renal biopsy-proven LN, proteinuria significantly decreased from 2.69 g/24 h at baseline to 0.51 g/24 h at 24 weeks, yet the CRR rates were only 48.9% and 56.3% at weeks 24 and 52, respectively (22). In a 1:1 matched cohort study, there was no significant difference in the CRR between the telitacicept and conventional treatment groups at 6 months, but the PERR was higher in the telitacicept group. At the end of follow-up (with a minimum follow-up of 6 months), there were significant differences in CRR (29.55% vs. 11.36%) and PERR (68.18% vs. 45.45%) between the telitacicept and conventional treatment groups (15). These differences in the CRR and PERR during follow-up may be attributable to variations in patient populations.

Consistent with the findings of previous studies (14–16, 22), telitacicept demonstrated significant reductions in the SLEDAI-2k score and increases in complement C3 and C4 levels over time, reflecting a decline in disease activity. Meanwhile, the IgG and IgA levels gradually decreased, indicating an improvement in immune hyperactivity. In our study, patients treated with add-on telitacicept had lower SLEDAI-2k scores than the control group at multiple time points (1, 3, 6, 9, and 12 months), whereas there were no significant differences in IgG, IgA, IgM, and complement C3 and C4 levels. Similarly, Jin et al.’s observational cohort study in patients with LN also found that the telitacicept group had lower SLEDAI-2k scores at the end of follow-up but no statistically significant differences in complement levels (15).In a cohort study targeting patients with class III–V LN, Chen et al. demonstrated that at 6 and 12 months, SLEDAI-2k scores were lower, and there were no significant differences in complement C3, C4, and IgA levels; however, the IgG and IgM levels were lower in the telitacicept group. Notably, in Chen et al.’s study, the baseline SLEDAI-2k scores of patients in the telitacicept group were higher than those in the control group, and baseline differences in the included patient populations may explain the discrepancies with our study findings (14).

Studies have shown that long-term, high-dose cumulative glucocorticoid use in patients with SLE is associated with adverse cardiovascular events, organ damage, and poor prognosis (23, 24). Reducing the glucocorticoid dosage is an important therapeutic goal in the management of SLE and LN. In our study, we observed a glucocorticoid-sparing effect of telitacicept, with 75% of patients in the telitacicept group having a glucocorticoid dosage of less than 5 mg/day at 6 months, significantly higher than the 20% in the control group; at 12 months, 70% of patients in the telitacicept group had a glucocorticoid dosage of less than 5 mg/day, significantly higher than the 35% in the control group. The overall trend of reduced glucocorticoid dosage with telitacicept compared to conventional therapy is consistent with previous reports on telitacicept treatment for SLE, both with and without LN (11, 14, 25).

We also found that although the renal relapse rate at 12 months was lower in the telitacicept group (30%) compared to the control group (45%), this difference did not reach statistical significance. However, the overall risk of relapse during the follow-up period was lower in the telitacicept group than that in the standard treatment group. Therefore, telitacicept may have long-term efficacy in preventing renal relapse, which requires further verification in randomized controlled trials.

In terms of safety, there were four cases (20.0%) of adverse reactions in the telitacicept group and seven cases (35.0%) in the standard treatment group, with no serious AEs reported in either group, suggesting that telitacicept add-on therapy did not increase AEs and might have a favorable safety profile in relapsed LN.

The question of which patients can benefit more from telitacicept treatment remains unanswered. Our findings demonstrated the efficacy of telitacicept in a specific patient population with relapsing LN. However, this study has some limitations. First, as a single-center retrospective study, it suffers from the common drawbacks of retrospective research such as missing data, selection bias, and loss to follow-up bias, which affect the generalizability of the results. Our study included only 40 patients, with a limited number of CRR and relapse events. In this context, the approach of using multivariable Cox regression may yield unstable hazard ratio estimates and overly narrow confidence intervals. Consequently, our findings should be considered hypothesis−generating and require external validation in larger independent cohorts. Second, by reviewing the previous treatment regimens of the included patients, it was impossible to standardize the types of immunosuppressants used in the standard treatment. Third, telitacicept for the treatment of LN is still in the clinical trial phase, and its application is limited compared to standard treatment regimens, resulting in a relatively small sample size. The limited renal pathology data, specifically the lack of biopsies in the telitacicept group, prevented us from correlating underlying pathology with prognosis. Additionally, changes in cells and cytokines such as CD19+ B cells, BAFF, and APRIL were not observed. In the future, well-structured randomized controlled trials and larger, multicenter, long-term studies will be needed to further evaluate the role of telitacicept in LN treatment and to define patient subgroups that are more suitable for its application.

## 5 Conclusion

As an add-on therapy, telitacicept is associated with early disease remission in relapsing lupus nephritis, which may suggest reduced disease activity, lower glucocorticoid dosage, fewer relapses, and no increase in AEs. Our study provides real-world evidence supporting the efficacy and safety of telitacicept in relapsed LN.

## Data Availability

The raw data supporting the conclusions of this article will be made available by the authors, without undue reservation.
