# Supplementary material for: Efficacy and safety of telitacicept as an add-on therapy for relapsing lupus nephritis: a retrospective cohort study
Source: Front Immunol. 2026 May 22;17:1826829. doi: 10.3389/fimmu.2026.1826829 (PMC13236884; doi:10.3389/fimmu.2026.1826829)
Supplement: Supplementary file 1 [file Table1.docx]

Supplementary Table1**.** Comparison of clinical characteristics and medications in censored and non-censored patients at baseline

|  | non-censored  (n=24) | censored  (n=16) | P |
| --- | --- | --- | --- |
| Group |  |  | <0.001 |
| Telitacicept | 19(95.0%) | 1(5.0%) | - |
| Standard therapy | 5(25.0%) | 15(75.0%) | - |
| Female, n (%) | 24(100%) | 16(100%) | - |
| age(y) | 35.42±13.30 | 28.81±11.80 | 0.116 |
| LN course(y) | 3.00(1,5.75) | 7.00(4.25,8.75) | 0.003 |
| relapse frequency |  |  | 0.665 |
| <2 | 18(75.0%) | 11(68.8%) | - |
| ≥2 | 6(25.0%) | 5(31.3%) | - |
| Hypertension(n%) | 9(37.5%) | 8(50.0%) | 0.433 |
| Hb (g/L) | 117.33±22.78 | 107.13±26.69 | 0.203 |
| PLT (10^9/L) | 221.62±85.85 | 183.81±84.33 | 0.177 |
| WBC (10^9/L) | 7.30(5.70,8.38) | 5.8(4.5,7.68) | 0.125 |
| Alb (g/L) | 34.60(30.13,39.30) | 24.25(20.55,30.90) | 0.011 |
| Scr (umol/L) | 61.50(50.50,74.45) | 102.00(71.00,207.50) | 0.003 |
| BUN (mmol/L) | 6.55(4.82,8.06) | 10.66(6.48,16.41) | 0.014 |
| eGFR(ml/min/1.73m^2) | 107.50(92.63,125.20) | 63.20(23.45,99.75) | 0.003 |
| Urine RBC(/HP) | 7.45（2.50，26.28） | 10.50（3.33,31.60） | 0.499 |
| 24h-UTP(g/24h) | 1.88(1.54，3.20) | 4.96(2.19,8.64) | 0.010 |
| UPCR (mg/mmol) | 256.71(169.14，395.42) | 565.80(263.72，1156.86) | 0.006 |
| Positive-Anti-Sm(n，%) | 9(37.5%) | 8(50.0%) | 0.433 |
| Positive-ANA≧1:320[n(%)] | 23(95.8%) | 16(100%) | - |
| Positive-anti ds-DNA[n (%)] | 15(62.5%) | 9(56.3%) | 0.693 |
| C3 (mg/dL) | 57.09±24.31 | 45.38±18.93 | 0.113 |
| C4 (mg/dL) | 10.45(7.00,15.35) | 7.00(5.35,12.13) | 0.109 |
| IgG (g/L) | 13.40(10.75,17.65) | 9.42(4.95,12.85) | 0.023 |
| IgA (g/L) | 2.88(1.87，3.60) | 2.01(1.42，3.29) | 0.116 |
| IgM (g/L) | 0.92(0.69，1.25) | 0.80(0.43，1.03) | 0.092 |
| SLEDAI-2K | 13.67±3.02 | 15.19±3.62 | 0.157 |
| glucocorticoid dosage(mg)/ day | 30(30.00, 50.00) | 40.00(30.00，50.00) | 0.229 |
| Pathology ISN/RPS class(%) | n=17 | n=15 | 0.148 |
| Ⅱ | 1(4.2%) | 1(6.3%) | - |
| Ⅲ/Ⅳ | 2(8.3%) | 5(31.3%) | - |
| Ⅲ/Ⅳ+Ⅴ | 10(41.7%) | 8(50.0%) | - |
| Ⅴ | 4(16.7%) | 1(6.3%) | - |
| no biopsy | 7(29.2%) | 1(6.3%) | - |
| Therapy |  |  | 0.187 |
| MMF | 14(58.3%) | 5(31.3.0%) | - |
| CTX | 2(8.3%) | 5(31.3%) | - |
| TAC | 4(16.7%) | 2(12.5%) | - |
| MMF+TAC | 4(16.7%) | 3(18.8%) | - |
| No | 0 | 1(6.3%) | - |
| LN, lupus nephritis; Hb, hemoglobin; PLT, platelet; WBC, white blood cell; Alb, albumin; UA, uric acid; BUN, blood urea nitrogen; Scr, serum creatinine; eGFR, estimated glomerular filtration rate; 24h-UTP, 24-h urinary total protein; UPCR, Urine protein creatinine rate; IgA, immunoglobulin A; IgG, immunoglobulin G; IgM, immunoglobulin M; SLEDAI-2K, The Systemic Lupus Erythematosus Disease Activity Index 2000;GC, glucocorticoids; MMF, mycophenolate mofetil; CTX, cyclophosphamide; TAC, Tacrolimus. | | | |

| Supplementary Table 2. Comparison of clinical characteristics and medications in patients treated with or without telitacicept at baseline, 1, 3, 6, 9, 12 months. | | | | | | | | | |
| --- | --- | --- | --- | --- | --- | --- | --- | --- | --- |
|  | Baseline | | | 1 month | | | 3 months | | |
| Group/time | Telitacicept | Standard therapy | P | Telitacicept | Standard therapy | P | Telitacicept | Standard therapy | P |
| Hb (g/L) | 115.45±25.91 | 111.05±23.70 | 0.578 | 122.10±16.97 | 117.15± 21.71 | 0.0427 | 126.20 ± 19.56 | 122.65 ± 22.80 | 0.600 |
| PLT (10^9/L) | 204.00±87.15 | 209.00±87.47 | 0.857 | 211.15 ± 95.61 | 242.45 ± 120.30 | 0.368 | 216.15 ± 86.11 | 260.0（198.25, 280.50） | 0.188 |
| Alb (g/L) | 32.67±7.73 | 28.08±7.81 | 0.069 | 37.73±5.18 | 33.60±7.31 | 0.046* | 39.48±3.48 | 38.95（29.18，42.45） | 0.461 |
| Scr (umol/L) | 63.50（53.25,78.50） | 83.50(52.50,191.00) | 0.172 | 61.5(55.00,69.50) | 68.50(60.25,133.00) | 0.053 | 58.00(52.00,69.00) | 70(58.75,138.75) | 0.031* |
| eGFR | 103.50(85.10,123.50) | 81.25(29.30,117.15) | 0.123 | 107.65(93.65,122.75) | 96.10(48.00,113.90) | 0.093 | 111.00(91.50,124.25) | 95.70(41.03,117.20) | 0.229 |
| 24h-UTP(g/24h) | 2.30（1.73，3.30） | 3.47（1.60，7.58） | 0.344 | 1.38（0.94，2.30） | 2.03（1.42，4.51） | 0.023 | 0.68（0.30，1.56） | 1.78（1.20，3.65） | 0.002 |
| UPCR（mg/mmol） | 294.50(169.14，395.42） | 394.64  （225.83，924.76） | 0.076 | 154.57（107.42，336.96） | 189.55  （150.86，522.59） | 0.152 | 85.13  （24.23，203.85） | 170.93  （119.37，298.82） | 0.013 |
| Urine RBC | 7.45（2.83，21.78） | 8.50（2.50，31.6） | 0.607 | 6.25(1.85,13.95) | 5.35(1.80,20.18) | 0.665 | 5.15(1.90,8.60) | 4.65(1.48,8.98) | 0.787 |
| ANA≧1:80[n（%）] | 19（95.0%） | 20（100%） | 1 | 16（80%） | 16（80%） | - | 14（70%） | 18（90%） | 0.236 |
| Anti-ds-DNA positive，  n (%)） | 12（60%） | 12（60%） | 1 | 8（40%） | 12（60%） | 0.343 | 3（15%） | 7（35%） | 0.273 |
| C3 (mg/dL) | 10.79±4.82 | 7.00（5.35，13.63） | 0.265 | 71.06 ± 12.01 | 69.00（49.40，82.18） | 0.279 | 70.80（66.55，81.25） | 80.20(61.18，90.75） | 0.051 |
| C4 (mg/dL) | 54.42±20.95 | 43.30（31.48，65.50） | 0.387 | 15.75(11.25,20.10) | 15.00(10.50,17.80) | 0.425 | 16.20（12.85，19.75） | 20.95(12.25,15.25) | 0.417 |
| IgG (g/L) | 12.96±5.02 | 11.35（7.18，14.00） | 0.372 | 10.96 ± 5.26 | 9.35 ± 5.65 | 0.355 | 9.98±4.81 | 8.58±4.21 | 0.337 |
| IgA (g/L) | 2.88（1.87，3.80） | 2.15（1.56，3.52） | 0.250 | 2.50（1.68，2.80） | 1.83（1.19，2.08） | 0.083 | 1.78（1.42，3.20） | 1.44（1.18，2.62） | 0.250 |
| IgM (g/L) | 0.92（0.63，1.20） | 0.80（0.45，1.15） | 0.358 | 0.63（0.31，0.78） | 0.62（0.32，1.01） | 0.490 | 0.44（0.25，0.60） | 0.49（0.34，0.96） | 0.250 |
| SLEDAI-2K score | 13.80±2.86 | 14.75±3.73 | 0.371 | 9.50±2.65 | 11.40±3.17 | 0.046 | 6.40±2.50 | 8.70±2.99 | 0.012 |
| glucocorticoid dosage  (mg)/ day | 30.00(30.00, 47.50) | 40.00(30.00，50.00) | 0.064 | 17.50 (11.25, 25.00) | 30.00 (21.25, 40.00) | <0.001 | 7.50 (5.00, 14.38) | 25.00 (10.00, 40.00) | <0.001 |
| Therapy |  |  | 0.612 |  |  | 0.612 |  |  | 0.631 |
| MMF | 10(50.0%) | 9(45.0%) | - | 10(50.0%) | 9(45.0%) | - | 10(50.0%) | 10(50.0%) | - |
| CTX | 2(10.0%) | 5(25.0%) | - | 2(10.0%) | 5(25.0%) | - | 2(10.0%) | 5(25.0%) | - |
| TAC | 4(20.0%) | 2(10.0%) | - | 4(20.0%) | 2(10.0%) | - | 4(20.0%) | 2(10.0%) | - |
| MMF+TAC | 4(20.0%) | 3(15.0%) | - | 4(20.0%) | 3(15.0%) | - | 4(20.0%) | 3(15.0%) | - |
| No | 0 | 1(5.0%) | - | 0 | 1(5.0%) | - | 0 | 0 | - |

|  | 6 months | | | 9 months | | | 12 months | | |
| --- | --- | --- | --- | --- | --- | --- | --- | --- | --- |
| Group/time | Telitacicept | Standard therapy | P | Telitacicept | Standard therapy | P | Telitacicept | Standard therapy | P |
| Hb (g/L) | 117.95 ± 19.74 | 122.65 ± 20.00 | 0.459 | 116.25 ± 24.95 | 122.55 ± 21.95 | 0.402 | 122.70 ± 16.48 | 122.35 ± 18.04 | 0.949 |
| PLT (10^9/L) | 223.12 ± 87.67 | 234.66 ± 70.44 | 0.649 | 224.89 ± 97.07 | 245.90 ± 82.91 | 0.466 | 209.45 ± 86.77 | 237.65 ± 65.46 | 0.253 |
| Alb (g/L) | 39.12±3.70 | 40.60（36.95，43.18） | 0.570 | 41.06±4.33 | 39.62±5.53 | 0.365 | 41.50(38.10,43.85) | 41.25(36.38,43.63) | 0.081 |
| Scr (umol/L) | 64.00（57.00，76.50） | 60.00（56.00，116.75） | 0.685 | 55.50(49.50,70.50) | 62.00(55.25,111.00) | 0.189 | 52.50（48.25,57.75) | 64.50(56.00,104.00)) | 0.050* |
| eGFR | 107.15(76.05,121.75) | 108.20(55.83,121.28) | 0.655 | 111.80(88.05,127.48) | 105.40(60.64,124.46) | 0.490 | 114.95(97.33,128.90) | 104.40(62.58,122.40) | 0.144 |
| 24h-UTP(g/24h) | 0.43（0.30，0.93） | 1.07（0.66，2.78） | 0.004* | 0.35（0.16，0.67） | 1.13（0.51，2.32） | 0.006* | 0.56(0.23,1.45) | 0.79(0.33,2.44) | 0.038* |
| UPCR（mg/mmol） | 50.28（22.41，105.59） | 142.32（72.07，285.03） | 0.005^*^ | 37.74(17.80，82.07) | 124.54(62.59，328.58） | 0.001* | 73.63(28.90,140.85) | 133.54(42.79,313.14) | 0.015* |
| Urine RBC | 3.85(1.80,6.58) | 3.10(1.80,5.50) | 0.745 | 3.25(1.65,7.38) | 2.45(0.23,6.35) | 0.456 | 2.85(0.53,6.28) | 2.40(0.30,8.03) | 0.918 |
| ANA≧1:80[n（%）] | 11（55%） | 19（95%） | 0.011* | 11(55%) | 15(75%) | 0.185 | 9/20(45%) | 15/20(75%) | 0.053 |
| Anti ds-DNA positive  [n（%）] | 5（25%） | 10（50%） | 0.102 | 7(35%) | 13(65%) | 0.058 | 7/20(35%) | 10/20(50%) | 0.337 |
| C3 (mg/dL) | 77.77±15.78 | 87.23±23.60 | 0.145 | 75.40±16.74 | 61.80±17.50 | 0.016* | 75.87±20.72 | 68.67±16.47 | 0.088 |
| C4 (mg/dL) | 16.00（12.93，19.35） | 17.00（16.00，25.75） | 0.228 | 18.00(11.75,25.40) | 16.50(11.05,20.00) | 0.386 | 13.00(10.00,16.00) | 19.00(15.00,27.75) | 0.924 |
| IgG (g/L) | 8.65（6.24，11.48） | 9.26±4.16 | 0.946 | 9.07±4.26 | 10.96±4.30 | 0.170 | 10.05±3.81 | 10.65±3.23 | 0.276 |
| IgA (g/L) | 1.18（0.92，2.53） | 1.37（1.04，2.38） | 0.598 | 1.20(0.82,2.34) | 1.50(1.28,2.16) | 0.190 | 1.28(1.16,2.34) | 1.98(1.46,2.13) | 0.120 |
| IgM (g/L) | 0.42（0.22，0.74） | 0.60（0.24，1.08） | 0.284 | 0.57(0.34,0.75) | 0.63(0.33,1.08) | 0.570 | 0.63±0.33 | 0.81±0.43 | 0.677 |
| SLEDAI-2K score | 5.55±2.93 | 7.30±3.63 | 0.048^*^ | 2.00(2.00,6.00) | 8.00(4.50,4.50) | 0.074 | 5.2±2.24 | 7.17±3.86 | 0.008* |
| glucocorticoid dosage  (mg)/ day | 5.00 (5.00, 6.88) | 10.00 (7.50, 20.00) | <0.001^*^ | 5.00(2.50,5.00) | 15.00(7.50,20.00) | <0.001* | 5.00(2.50,10.00) | 15(5.00,23.75) | 0.007* |
| Therapy |  |  | 0.386 |  |  | 0.543 |  |  | 0.433 |
| MMF | 13(65.0%) | 10(50.0%) | - | 12(60.0%) | 9(45.0%) | - | 11(60.0%) | 9(45.0%) | - |
| CTX | 0(0.0%) | 0(0.0%) | - | 0(0.0%) | 0(0.0%) | - | 0(0.0%) | 0(0.0%) | - |
| TAC | 4(20.0%) | 3(15.0%) | - | 3(15.0%) | 2(10.0%) | - | 4(15.0%) | 2(10.0%) | - |
| MMF+TAC | 3(15.0%) | 7(35.0%) | - | 5(25.0%) | 9(45.0%) | - | 5(25.0%) | 9(45.0%) | - |

LN, lupus nephritis; Hb, hemoglobin; PLT, platelet; Alb, albumin; UA, uric acid; BUN, blood urea nitrogen; Scr, serum creatinine; eGFR, estimated glomerular filtration rate; 24h-UTP, 24-h urinary total protein; UPCR, UPCR, Urine protein creatinine rate ; IgA, immunoglobulin A; IgG, immunoglobulin G; IgM, immunoglobulin M; SLEDAI-2K, The Systemic Lupus Erythematosus Disease Activity Index 2000; MMF, mycophenolate mofetil; CTX, cyclophosphamide; TAC, Tacrolimus.
